# Ofatumumab for the treatment of refractory anti‐LGI1 encephalitis with long‐term poor blood glucose control in type 1 diabetes

**DOI:** 10.1111/cns.14416

**Published:** 2023-08-21

**Authors:** Kaili Chen, Le Yang, Lei Xu, Yan Jiang, Jinting He

**Affiliations:** ^1^ Department of Neurology China‐Japan Union Hospital of Jilin University Changchun China; ^2^ Department of Endocrinology Jilin Province People's Hospital Changchun China

1

## 1 INTRODUCTION

Ofatumumab (OFA) has been approved for the treatment of multiple sclerosis, works by depleting B lymphocytes, and has shown positive results.^1^ Ofatumumab, a recombinant human monoclonal immunoglobulin G1 antibody that binds to B‐cell expressed human CD20, may have potential in the treatment of autoimmune encephalitis and type 1 diabetes. We report a rare case of refractory anti‐LGI1 encephalitis with long‐term poor blood glucose control in a patient with type 1 diabetes. After OFA treatment (20 mg/time), we found that CD20^+^ and CD19^+^ lymphocytes were depleted, as well as a significant improvement in cognition, excessive daytime drowsiness and blood glucose levels, no further hyponatremia, and no significant adverse effects. Thus, our study suggests that OFA is effective and safe as a novel anti‐CD20 monoclonal antibody for the treatment of refractory anti‐LGI1 encephalitis with long‐term poor blood glucose control in type 1 diabetes.

## 2 CASE REPORT

**FIGURE 1:**
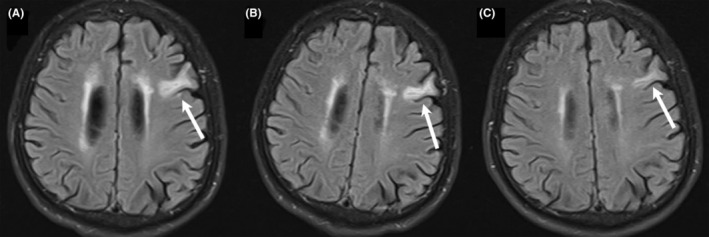
T2 FLAIR images from the patient's cranial MRI evaluation. (A–C) show the images at the time of first diagnosis, the time of recurrence, and the time after the use of OFA for 1 month, respectively.

A 62‐year‐old man was admitted in July 2022, with presenting symptoms of episodic convulsions, memory decline, and excessive daytime drowsiness 3 months prior to admission. Two seizures occurred within 2 weeks before he was admitted to the hospital; the seizures manifested as involuntary twitching of his right hand, which lasted approximately 3–5 s, followed by flexion of both upper limbs, ankylosis of both lower limbs, unconsciousness, and upward movement of the eyes, which lasted for approximately 5 min. He was previously healthy, with no history of any disease except type 1 diabetes and no family history of inherited diseases. Initial examination showed drowsiness, impaired consciousness, and mixed‐type aphasia, with a Modified Rankin Scale (mRS) score of 4, a Mini‐Mental State Examination (MMSE) score of 2/30, and a Montreal Cognitive Assessment (MoCA) score of 2/30. Brain MRI demonstrated dotted and lamellar abnormal signals in the left frontal lobe, with a low signal in T1, high signal in T2, and high signal in T2 Flair images (Figure 1A). Laboratory examination revealed a positive result for sodium (123.4 mmol/L), fasting blood glucose (FBG) 10.40 mmol/L, glycosylated hemoglobin 9.5%, urine ketone bodies 1+, and C‐peptide level <0.20 ng/mL. Tests for antileucine‐rich glioma inactivated 1 (anti‐LGI‐1) antibody were positive in CSF (titer 1:10) and serum (titer 1:10) samples. Blood pressure, electrocardiogram, bilateral cervical vascular ultrasound, cardiac ultrasound, and arteriovenous ultrasound of the extremities did not show any significant abnormalities. Genetic testing was performed on the patient and his son; whole‐exome sequencing revealed that the patient was an HLA DRB1*07:01 carrier, while the susceptibility genes of theirs associated with type 1 diabetes were negative. Based on these findings, he was diagnosed with anti‐LGI1 encephalitis with type 1 diabetes.

After a total of five repeated rounds of immunoglobulin treatment, no significant improvement in symptoms or signs was observed in the patient; in fact, his condition worsened. In January 2023, the patient's cognitive dysfunction and excessive daytime drowsiness further worsened, and he slept for up to 18 hours per day and exhibited no significant cycling of sleep. Examination showed an mRS score of 4, an MMSE score of 3/30, an MoCA score of 2/30 and positivity for serum anti‐LGI1 antibodies (1:10). The brain MRI results are shown in Figure 1B. Due to the failure of conventional immunization treatment, it was decided that the treatment would be changed. Therefore, we gave the patient a single subcutaneous OFA injection (20 mg), and no significant adverse effects were observed after the injection.

**FIGURE 2:**
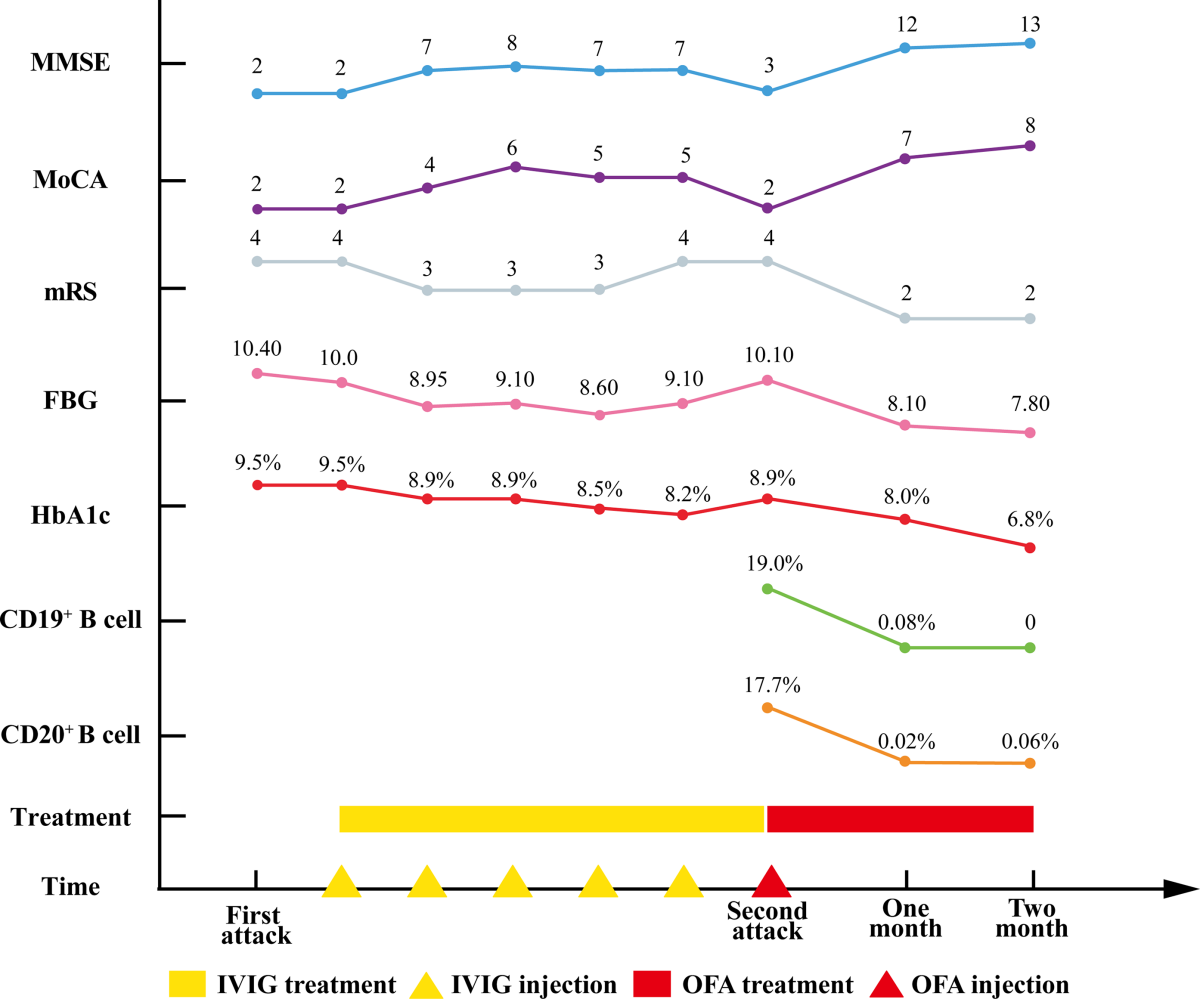
Changes in the MMSE score, MoCA score, mRS score, FBG level, HbA1c, CD19^+^ B‐cell percentage, CD20^+^ B‐cell percentage, and treatment details of the case.

After 1 month of using Ofatumumab, the patient had significantly improved, and brain MRI showed that the lesion was smaller than it had been on previous scans (Figure 1C). The patient's blood glucose level was better controlled, despite a reduction in his insulin dose reduced, and there were no significant abnormalities in his blood sodium levels. His cognitive dysfunction and excessive daytime sleepiness improved. Two‐month follow‐up period after OFA treatment, the patient's symptoms improved significantly, and no significant adverse effects were observed. Further details on the disease course are summarized in Figure 2. The patient provided written informed consent, and this study was approved by the Ethics Committees of China‐Japan Union Hospital of Jilin University (No. 2023070310).

## 3 DISCUSSION

This is the first report to investigate the potential of OFA for the treatment of refractory anti‐LGI1 encephalitis with long‐term poor blood glucose control in a patient with type 1 diabetes. Our case report demonstrates the efficacy and safety of OFA and suggests the possibility of onset and treatment relevance to both autoimmune diseases.

Anti‐LGI1 encephalitis and type 1 diabetes are inflammatory diseases, and a study showed that one in every five people with type 1 diabetes also has another type of autoimmune disease due to shared genetic mechanisms and immune processes, and the more common autoimmune diseases include hypothyroidism, Addison's disease and coeliac disease.^2^ However, very few cases of autoimmune encephalitis combined with type 1 diabetes have been reported in the literature, and no cases of anti‐LGI1 autoimmune encephalitis combined with type 1 diabetes have been reported; thus, our case is relatively rare.

Studies have shown that humoral immunity is involved in the development of anti‐LGI1 encephalitis and type 1 diabetes; therefore, treatments targeting B cells are therapeutic.^3^, ^4^ The anti‐CD20 monoclonal antibody is an immunosuppressive agent that functions by triggering cell and complement‐mediated cytotoxicity, leading to the depletion of CD20^+^ B lymphocytes.^5^ Rituximab, an anti‐CD20 monoclonal antibody, has been used to treat type 1 diabetes and autoimmune encephalitis with proven efficacy.^6^, ^7^ After decades of pharmacological development, fully humanized anti‐CD20 monoclonal antibodies have been produced. Ofatumumab is one such antibody targeting CD20 that has fewer adverse effects than rituximab. Studies have shown that OFA has stronger binding affinity to CD20, a slower dissociation rate, and better efficacy than rituximab and is safer and more convenient in clinical applications because it is administered subcutaneously.^8^ In addition, it has shown superior results to those of traditional immunotherapy in the treatment of multiple sclerosis and neuromyelitis optica spectrum disorder.^1^, ^9^ Therefore, OFA is theoretically effective for the treatment of autoimmune encephalitis with type 1 diabetes when conventional immunotherapy has failed. In addition, our study validated the genetic association between HLA‐DRB1*07:01 and anti‐LGI1 encephalitis, providing evidence for a possible genetic mechanism for the development of autoimmune encephalitis.^10^

This study is a relatively rare case of anti‐LGI1 autoimmune encephalitis combined with type 1 diabetes mellitus, in which conventional immune treatment was ineffective but the prognosis was positive after treatment with OFA. Therefore, timely treatment with OFA may be a clinically beneficial option for patients with autoimmune encephalitis when conventional immunotherapy is ineffective, representing a new and effective treatment strategy for severe antibody‐mediated autoimmune encephalitis, especially in combination with other autoimmune diseases. More observational studies with large samples are needed in the future to further verify the efficacy and safety of OFA for the treatment of this type of disease.

## 4 AUTHOR CONTRIBUTIONS

Kaili Chen assessed the patient, performed the review of the literature, and drafted the manuscript and figures. Le Yang performed the review of the literature, assisted with genetic testing, and drafted the manuscript and figures. Jinting He assessed the patient, revised the manuscript and contributed to supervision, funding acquisition, and project administration. Lei Xu assessed and treated the patient. Yan Jiang performed the review of the literature and edited the manuscript for important intellectual content.

## 6 CONFLICT OF INTEREST STATEMENT

The authors declare no conflicts of interest.

## Data Availability

The data that support the findings of this study are available from the corresponding author upon reasonable request.
